# Mechanisims of asthma and allergic disease – 1070. Genetic mechanisms of immune imbalance in allergic inflamation

**DOI:** 10.1186/1939-4551-6-S1-P67

**Published:** 2013-04-23

**Authors:** Uktam Ziyadullaev

**Affiliations:** 1 Obstetrics and Gynecology, Haitova N.M., Khamdamov A.M., Ziyadullaev.Sh.Kh, Tashkent, Uzbekistan

## Background

At the present time is considered an established fact that at every stage of the immunological response to free radicals and their derivatives, antioxidant enzymes perform a regulatory function and may help limit or activation of immune responses. In this regard, of interest to identify the functionally significant variant genotype polymorphism Ile-105/Val-105 GSTπ1, which can contribute to and interfere with the functioning of the immune system in allergic diseases.

## Methods

A total of 91 patients with various forms of allergic diseases and healthy individuals of Uzbek nationality. Verification of the diagnosis of allergic diseases in various organs and tissues was carried out according to the WHO International Classification (ICD-10). Analysis of the polymorphic variants of the gene GSTπ1 was performed by polymerase chain reaction of DNA synthesis (PCR) thermocycler and RFLP analysis followed by polyacrylamide gel electrophoresis. Determination of lymphocyte immunophenotype CD3^+^, CD4^+^, CD8^+^, CD16^+^, CD19^+^, CD23^+^, CD95^+^performed with monoclonal antibodies and the level of Ig classes A, M, G by radial immunodiffusion.

## Results

In our studies revealed that in the group with genotype Ile/Ile patients registered declines in CD3^+^, CD8^+^ and CD16^+^ up to 33,6 ± 0,45%, 15,6 ± 0,88% and 15,6 ± 0,88%, respectively compared with a group of people who did not have this genotype. Registered a significant increase in IgG level in patients with allergic inflammation, with the genotype homozygous and heterozygous genotype Ile-105/Ile-105 Ile-105/Val-105 compared with the homozygous genotype Val-105/Val-105 (p<0,01 in both cases). Expressed lower levels of IgA was associated with the presence of genotype I/I patients (p<0,05). Significant differences are manifested decreased content of CD95^+^, depending on the gene polymorphism Ile-105/Val-105 GSTπ1 studied patients reported in patients with genotype Ile-105/Ile-105 compared with the group with the genotype Ile-105/Val-105 (p<0,05).

## Conclusions

Thus, the identified association Ile-105/Val-105 GSTπ1 gene polymorphism with indices of immune status in allergic diseases indicates that the genotype Ile-105/Ile-105 GSTπ1 gene plays an important role in the formation of immunological disorders in the pathogenesis of allergic diseases.

